# Laparoscopic Pectopexy: A Biomechanical Analysis

**DOI:** 10.1371/journal.pone.0144143

**Published:** 2016-02-04

**Authors:** A. Sauerwald, M. Niggl, J. Puppe, A. Prescher, M. Scaal, G. K. Noé, S. Schiermeier, M. Warm, C. Eichler

**Affiliations:** 1 Department of Gynecology and Obstetrics, Hospital Düren GmbH, Düren, Germany; 2 Breast Cancer Center, Municipal Hospital Holweide, Cologne, Germany; 3 Department of Gynecology and Obstetrics, Municipal Hospital Holweide, Cologne, Germany; 4 Department of Gynecology and Obstetrics, University of Cologne, Cologne, Germany; 5 Department of Anatomy, RWTH Aachen University, Aachen, Germany; 6 Department of Anatomy II, University of Cologne, Cologne, Germany; 7 Dep. Ob/Gyn Hospitals Rhein-Kreis-Neuss, Faculty University of Witten/Herdecke, Witten, Germany; 8 Dep. Ob/Gyn Marienhospital Witten, Witten, Germany; University of Insubria, ITALY

## Abstract

**Introduction:**

Pectopexy, a laparoscopic method for prolapse surgery, showed promising results in recent literature. Further improving this approach by reducing surgical time may decrease complication rates and patient morbidity. Since laparoscopic suturing is a time consuming task, we propose a single suture /mesh ileo-pectineal ligament fixation as opposed to the commonly used continues approach.

**Methods:**

Evaluation was performed on human non-embalmed, fresh cadaver pelves. A total of 33 trials was performed. Eight female pelves with an average age of 75, were used. This resulted in 16 available ligaments. Recorded parameters were ultimate load, displacement at failure and stiffness.

**Results:**

The ultimate load for the mesh + simplified single “interrupted” suture (MIS) group was 35 (± 12) N and 48 (± 7) N for the mesh + continuous suture (MCS) group. There was no significant difference in the ultimate load between both groups (p> 0.05). This was also true for displacement at failure measured at 37 (± 12) mm and 36 (±5) mm respectively. There was also no significant difference in stiffness and failure modes.

**Conclusion:**

Given the data above we must conclude that a continuous suture is not necessary in laparoscopic mesh / ileo-pectineal ligament fixation during pectopexy. Ultimate load and displacement at failure results clearly indicate that a single suture is not inferior to a continuous approach. The use of two single sutures may improve ligamental fixation. However, overall stability should not benefit since the surgical mesh remains the limiting factor.

## Introduction

In 2010 a new laparoscopic method for prolapse surgery, especially designed for obese women, called pectopexy, was presented by Noe et al. [1]. Literature suggests abdominal sacrocolpopexy to still be the gold standard [2–5]. However, laparoscopic sacrocolpopexy and robot-assisted sacrocolpopexy offer, although more cost and time consuming, effective alternatives [4,6]. In cases where it is difficult to reach the os sacrum however, a pectopexy has also been discussed as a useful and very effective option in recent literature [1,7–9]. The use of the ileo-pectineal ligament for a tension free mesh suspension is not a new concept and has been used by other groups [9,10]. Given the potential of this new method, we find that pectopexy is a surgical approach which may further be optimized. This is especially true since recent results from a prospective, randomized, clinical trial comparing laparoscopic sacrocolpopexy to laparoscopic pectopexy showed the latter to not reduce pelvic space, resulting in, a zero percentage of defecation disorders (compared to 19.5% in the sacrocolpopexy group) [8]. Literature describes the exact pectopexy fixation method of the transverse mesh as being performed with 2 separate stitches on the ileo-pectineal ligament with nonabsorbable sutures (No. 0 with attached needle) [7]. Noe et al. generally uses two single “interrupted” sutures or, in case of a colposuspension, a “continuous” type suture. While most of the surgical steps as described by Banerjee et al. [1] and Alkatout et al. [7] may not be eliminated, surgical time may only be optimized by omitting unnecessary procedures. Since laparoscopic continuous suturing is time-consuming we hypothesized that a single suture may also be sufficient. In order to evaluate this concept a randomized clinical trial should be initiated. Any deviation from gold standard should however be first supported with some sort of biomechanical data; this study therefore asks the following questions:

1. What load may be supported by standard (continuous) ileo-pectineal fixation methods?
2. May this load be supported by a single “interrupted” suture?
3. What are the biomechanical properties of the ileo-pectineal mesh /suture fixation?
4. What is the limiting factor of said fixation method (mesh, ligament, suture)?

## Methods

**Fig 1:**
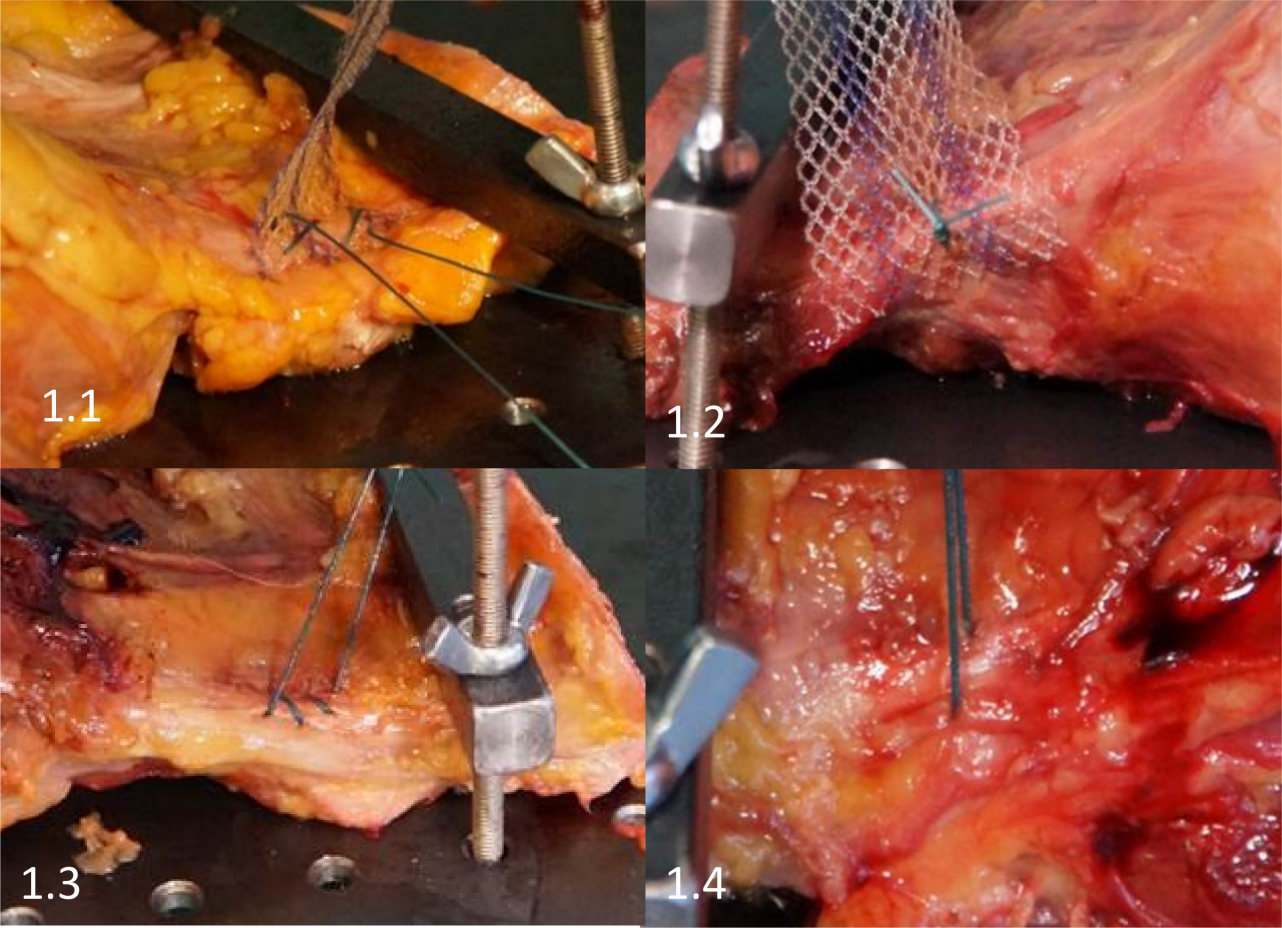
Shown are representative images of the 4 evaluated groups. Fig 1.1 shows mesh + continuous suture (MCS). Fig 1.2 shows mesh + a single interrupted suture (MIS). Fig 1.3 shows a continuous suture (CS) and Fig 1.4 shows a single interrupted suture (IS).

Fixation method evaluation was performed on human non-embalmed, fresh cadaver pelves. Preparation of the ileo-pectineal ligament was performed by an experienced surgeon. Eight female pelves, average age 75, were used. This resulted in 16 available ligaments. All cadavers were procured from the Institute of Anatomy at the University of Aachen. Identifying data was available only to coauthor P.A. A total of 33 trials were performed. We were able to retest ligaments since all initial trials resulted in mesh failure at loads well within the elastic region of the load-displacement diagram. 4 types of trials were conducted. Group 1 (n = 9) used a single “interrupted” suture in combination with a mesh (MIS). One additional trial run resulted in data that could, unexpectedly, be included into this group. Group 2 (n = 8) evaluated the same mesh and a continuous suture (MCS). Group 3 (n = 8) evaluated a single “interrupted” suture (IS) and group 4 (n = 8) assessed continuous sutures (CS). An undyed, monofilament, partially absorbable mesh was used: SERA MESH® PA (15 x 5 mm—SERAG-WIESSNER GmbH & Co. KG, Naila, Germany). A synthetic, braided, non-absorbable Ethibond suture 0, FSLX needle, 75 cm green filament (Ethicon / Johnson & Johnson, Somerville, NJ, USA) was used in all 4 groups. Fig 1 shows all 4 fixation methods.

Analysis was performed on an Instron 5565^®^ test frame using the Bluehill 2 Software^®^. All tests were transient evaluations of the individual fixation methods at 5 N/s load increase. Recorded parameters were ultimate load (N) and displacement at failure (mm). These resulted in calculated parameters such as stiffness (N/mm) and load at 2 mm displacement. The latter being considered as fixation failure in biomechanical evaluations since stability may be lost as dehiscence exceeds 2 mm. [11–15]

### Procedure

Cadaver pelves were prepared in a manner allowing the ileo-pectineal ligament to be placed in the Instron 5565^®^ test frame appropriately, while still attached to the surrounding bone structure. Cadavers were only unfrozen once. Testing proceeded in a continuous /transient manner thereafter. As shown in Fig 1.1 the continuous suture + mesh group allowed for a fixation of the mesh on the ileo-pectineal ligament in a manner allowing for large areas of the mesh to be covered by suture material. Fig 1.2 shows a single “interrupted” suture attaching the mesh to the ligaments. Fig 1.3 shows above-mentioned continuous suture attaching to the ligament without a mesh and Fig 1.4 shows a single suture loop around the ileo-pectineal ligament. 3 mesh-loops were covered in the single “interrupted” suture, allowing adequate suture/mesh surface interaction. At least a 2-loop safety margin was maintained to the mesh edge. Suture length between ligament and the test frame was the same in all experiments. While suture distortion factored into all results, this effect was the same for all trials and may therefore be omitted.

### Statistics

Statistical analysis was performed using the VassarStats^®^ (Vassar College, Poughkeepsie, NY, USA) statistics program. ANOVA analysis and t-tests were used in order to evaluate significances when appropriate.

### Ethics Committee Approval

This study was conducted in accordance with institutional review board standard operating procedures. An ethics committee vote was initiated, but deemed unnecessary by the “Ethikkommission der Aerztekammer Nordrhein”. A written statement to this extent is available.

## Results

**Fig 2:**
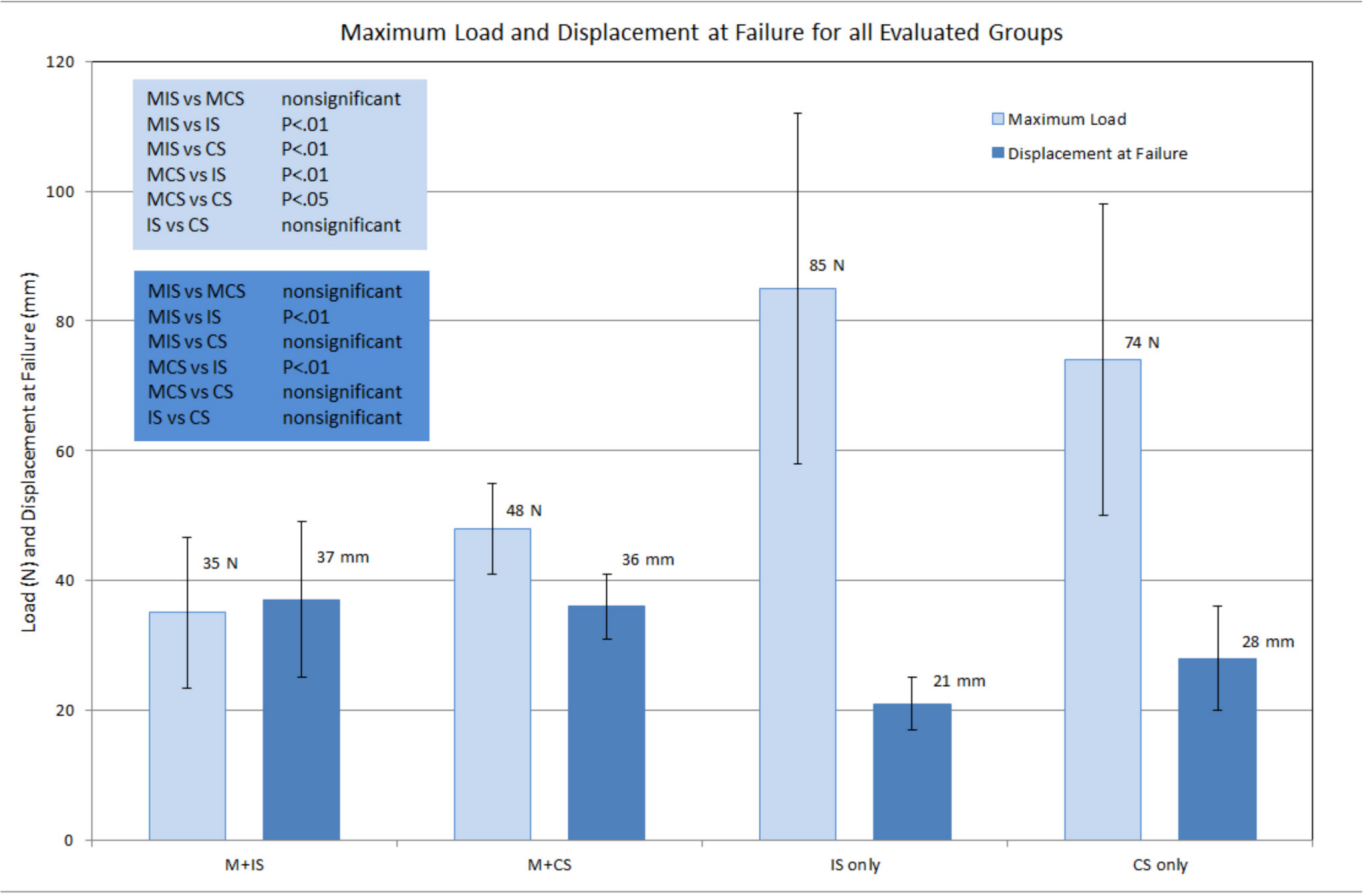
Shown are maximum loads and displacement at failure for all four subgroups. ANOVA analysis results are shown as well. Error bars represent standard deviations. M+IS = mesh + interrupted (single) suture, M + CS = mesh + continuous suture, IS = interrupted (single) suture, CS = continuous suture.

**Fig 3:**
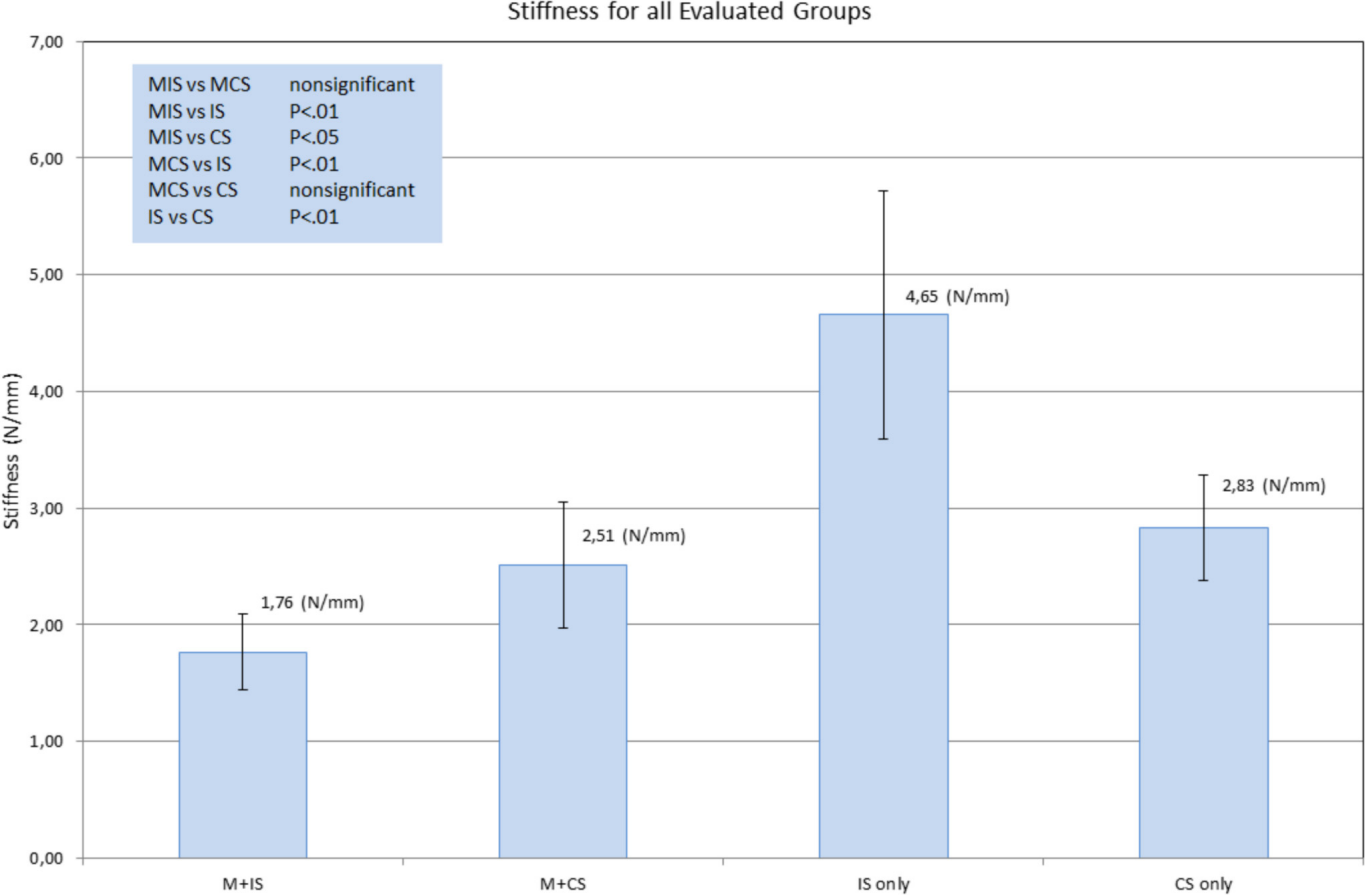
Shown is fixation method stiffness for all four subgroups. ANOVA analysis results are shown as well. Error bars represent standard deviations. M+IS = mesh + interrupted (single) suture, M + CS = mesh + continuous suture, IS = interrupted (single) suture, CS = continuous suture.

A total of 33 trials was conducted, resulting in four subgroups. A summary of all results is given in Table 1. Due to the complexity of intergroup ANOVA analyses, p values are listed in Figs 2 and 3.

**Table 1:** This table show overall results for all four evaluated groups.

Evaluated Entity	Number Tested	Ultimate Load	Displacement at Failure	Stiffness	Calculated Load at 2 mm	Failure Mode
Total Trials = 33	n	N	mm	N /mm	N	
Mesh + Interrupted Suture	9	35 (± 12)	37 (± 12)	1,76 (± 0.32)	3.5 (± 0.6)	Individual loop failure (9/9)
Mesh + Continuous Suture	8	48 (± 7)	36 (± 5)	2,51 (± 0.53)	5.0 (± 1.1)	Global mesh failure (8/8)
Only Interrupted Suture	8	85 (± 27)	21 (± 4)	4,65 (± 1.06)	9,3 (± 2.1)	Ligament failure (5/8) Suture failure (3/8)
Only Continuous Suture	8	74 (± 24)	28 (± 8)	2,83 (± 0.46)	5.6 (± 0.9)	Ligament failure (3/8) Suture failure (5/8)

Group 1 represents the hypothesis group with mesh + single “interrupted” suture and group 2 represents the gold standard mesh + continuous suture. Both groups showed the mesh to be the limiting factor. The number of times the sutured was threaded through the surgical mesh was irrelevant. Neither the suture nor the ileo-pectineal ligament failed in all 17 trials (groups 1 and 2). The ultimate load for the MIS group was 35 ± 12 N and 48 ± 7 N for the MCS group. There was no significant difference in the ultimate load between both groups (p> 0.05). The same holds true for displacement at failure which was 37 ± 12 mm (MIS) and 36 ±5 mm (MCS). There is also no significant difference in stiffness between the single suture group and continuous suture group. The only difference wasthe method of failure. The single suture + mesh group resulted in a failure where suture material would cut through an individual loop of the applied mesh. This was not the case for the continuous suture group. The latter showed global mesh deterioration. This, although different, did not result in a significant difference in maximum load, displacement at failure and fixation method stiffness.

Test groups 3 and 4 evaluated the suture techniques on their own. This, representing a control group, resulted in either ligament failure or suture tearing. Having eliminated the limiting factor “mesh”, failure loads were significantly higher 85 ± 27 N for the single “interrupted” suture group and 74 ± 24 N for the continuous suture group. Failure loads without the mesh did not differ significantly between suture methods (see Fig 2), neither did displacement at failure. Interestingly, a significant difference could be shown for fixation method stiffness where the individual suture showed to be significantly stiffer than all tests groups at 4.65 ± 1.06 N/mm (p > 0.01 –for all groups). The failure mode within groups 3 and 4 was either ligament failure or suture failure. Distribution of suture and ligament failure did not differ between single suture and continue suture groups.

## Discussion

To our knowledge there has thus far not been any biomechanical analysis performed in this specific area, although some data regarding pelvic ligament strength is available [16]. Overall, our results support the primary hypothesis that a single suture is sufficient in a pectopexy scenario. As shown in Table 1, ultimate load, fixation stiffness and displacement at failure do not differ significantly when comparing the continuous suture application to a single “interrupted” suture. This indicates that a continuous suture method is not superior to a single “interrupted” suture scenario. Alternatively, the use of two single sutures may be considered, but since the limiting factor seems to be the applied surgical mesh an additional single loop may not be beneficial. This is also supported by the failure modes listed in Table 1. We found that single “interrupted” sutures supported loads of up to 85 N. Since, the SERA MESH® PA failed well before this load was achieved we may either opt for a more sturdy mesh or accept the fact that the number and manner of sutures is not important for overall fixation method stability. Additionally, literature has not reported mesh failure in a pectopexy scenario and personal experience shows this not to be a problem in everyday clinical practice. We therefore argue that a single “interrupted” type suture is an adequate option for mesh fixation.

### Ultimate load

While it is important to understand that the limiting factor is the applied mesh, other factors regarding ultimate load analysis should also be addressed. A load of 35 N as reported in the MIS situation is considerable. Zimkowski et al. recently reported surgical polyester mesh (PETKM14001, Textile Development Associates) failure at 16.65 ± 3.30N [17] in a semi-physiological setting. Our results maintain the same order of magnitude, thus limited comparability is given. A direct comparison remains difficult since different materials were used. Results are nonetheless similar to those reported in literature and allow us to postulated study comparability to a physiological setting. Furthermore, issues such as immediate postoperative mesh-deformation, known from hernia repair [18], may certainly be prevented by a fixation method allowing for a load of at least 35 N. Since, literature has not reported biomechanical data for this specific method, larger trials may have to be initiated in order to clearly define the biomechanical spectrum for pectopexy.

### Displacement at failure

This parameter has been previously investigated in an in vitro situation, in an attempt to reduce surgical time [17,19]. When comparing our data to Zimkowski et al. (control mesh failure at approx. 21.92 ± 3.76 mm) our findings (36–37 mm) agree with literature. From a clinical point of view, a maximum of 37 mm of displacement seems adequate.

### Stiffness

The topic of fixation method stiffness is important. This parameter describes the elongation due to force of a given entity. Stiffness, being highly dependent on the trial setup, may not be compared to literature since this reported data is the first of its kind. We found stiffness to be lowest in the mesh groups. Suture methods did not impact this parameter significantly (within the mesh groups). However, a trend favoring the continuous suture may be observed here. The relevance of this fact is not yet known, especially since the “suture only” groups (no mesh) show the opposite to be true.

### Calculated Load at 2 mm Displacement

This parameter is directly proportional to the stiffness and serves only as a point of comparison to biomechanical analyses performed in recent literature. A 2 mm displacement is considered initial failure in orthopedics. While soft tissue surgery may allow for more error, the authors felt this value to be worth listing in order to improve inter-publication comparability.

The biomechanical analysis of a single suture versus a continuous suture with and without mesh used in a pectopexy scenario yielded the following results.

1. What load may be supported by standard (continuous) ileo-pectineal fixation methods? Loads of up to 48 N may be supported by the standard fixation method.
2. May this load be supported by a single “interrupted” suture? There is no statistically significant difference between continuous and single “interrupted” suture fixation methods. 2 single sutures may improve ligamental fixation, but overall stability does not benefit.
3. What are the biomechanical properties of the ileo-pectineal mesh /suture fixation? This is the first reported data in this area. Table 1 shows a wide array of biomechanical data on this subject.
4. What is the limiting factor of said fixation method (mesh, ligament, suture)? All tests lead to the conclusion that the limiting factor is the utilized surgical mesh.

### Further Investigation

Despite the obvious need for long term in-vivo testing and the ever present benefit of increased sample size, we feel the need to mention that variability in ligament quality may also play a role in everyday clinical practice and must not be ignored [16].

## Conclusion

Given the data above we must conclude that a continuous suture is not necessary in laparoscopic mesh / ileo-pectineal ligament fixation during pectopexy. Ultimate load and displacement at failure results clearly indicate that a single “interrupted” suture is not inferior to a continuous approach. The use of 2 single sutures may improve ligamental fixation. Theoretically, overall stability should not benefit since the surgical mesh remains the limiting factor.

## Supporting Information

S1 File Contains the data with the stress stain diagrams for the suture (without mesh) trial runs. (XLSX)

S2 File Contains the data with the stress stain diagrams for the suture with mesh trial runs. (XLSX)
